# Identification of Mammalian and Poultry Species in Food and Pet Food Samples Using 16S rDNA Metabarcoding

**DOI:** 10.3390/foods10112875

**Published:** 2021-11-20

**Authors:** Laura Preckel, Claudia Brünen-Nieweler, Grégoire Denay, Henning Petersen, Margit Cichna-Markl, Stefanie Dobrovolny, Rupert Hochegger

**Affiliations:** 1 Chemical and Veterinary Analytical Institute Muensterland-Emscher-Lippe (CVUA-MEL), Joseph-Koenig-Strasse 40, 48147 Muenster, Germany; laura.preckel@cvua-mel.de; 2 Chemical and Veterinary Analytical Institute Rhein-Ruhr-Wupper (CVUA-RRW), Deutscher Ring 100, 47798 Krefeld, Germany; gregoire.denay@cvua-rrw.de; 3 Chemical and Veterinary Analytical Institute Ostwestfalen-Lippe (CVUA-OWL), Westerfeldstrasse 1, 32758 Detmold, Germany; henning.petersen@cvua-owl.de; 4 Department of Analytical Chemistry, Faculty of Chemistry, University of Vienna, Währinger Strasse 38, 1090 Vienna, Austria; margit.cichna@univie.ac.at; 5 Austrian Agency for Health and Food Safety (AGES), Institute for Food Safety Vienna, Department for Molecular Biology and Microbiology, Spargelfeldstrasse 191, 1220 Vienna, Austria; stefanie.dobrovolny@ages.at (S.D.); rupert.hochegger@ages.at (R.H.)

**Keywords:** DNA metabarcoding, 16S rDNA, meat species identification, authentication, food, pet food, feed, real-time PCR, PCR array

## Abstract

The substitution of more appreciated animal species by animal species of lower commercial value is a common type of meat product adulteration. DNA metabarcoding, the combination of DNA barcoding with next-generation sequencing (NGS), plays an increasing role in food authentication. In the present study, we investigated the applicability of a DNA metabarcoding method for routine analysis of mammalian and poultry species in food and pet food products. We analyzed a total of 104 samples (25 reference samples, 56 food products and 23 pet food products) by DNA metabarcoding and by using a commercial DNA array and/or by real-time PCR. The qualitative and quantitative results obtained by the DNA metabarcoding method were in line with those obtained by PCR. Results from the independent analysis of a subset of seven reference samples in two laboratories demonstrate the robustness and reproducibility of the DNA metabarcoding method. DNA metabarcoding is particularly suitable for detecting unexpected species ignored by targeted methods such as real-time PCR and can also be an attractive alternative with respect to the expenses as indicated by current data from the cost accounting of the AGES laboratory. Our results for the commercial samples show that in addition to food products, DNA metabarcoding is particularly applicable to pet food products, which frequently contain multiple animal species and are also highly prone to adulteration as indicated by the high portion of analyzed pet food products containing undeclared species.

## 1. Introduction

Commercial food and feed products must meet the requirements of national and international regulations. Manufacturers have to ensure that their products are both safe and authentic. However, food fraud has become a global issue, with meat products being particularly vulnerable to adulteration [1]. The term food fraud encompasses a variety of activities that are committed intentionally and aimed at deceiving consumers with respect to food quality. Meat products are frequently found to be adulterated by substitution of animal species given on the label by animal species of lower commercial value [2].

Food controls play a crucial role in the mitigation of food fraud. For the differentiation of animal species in food products, various molecular methodologies have been developed, including protein- and DNA-based ones [2,3,4,5,6]. DNA-based methodologies make use of genetic variations between species, e.g., single nucleotide polymorphisms (SNPs), insertions and deletions. They target either species-specific fragments in nuclear DNA or conserved regions in the mitochondrial genome. At present, DNA arrays and real-time PCR assays are mainly used for the authentication of meat products in official food laboratories.

DNA arrays are based on DNA hybridization [7]. In a first step, the target region, e.g., a conserved region of 16S rDNA, is amplified using biotinylated primers, resulting in the formation of biotinylated PCR products. The labeled PCR products are hybridized to species-specific oligonucleotide probes prespotted on a chip. After removing unbound PCR products by washing, hybridized PCR products are detected enzymatically. Commercial DNA arrays for animal species differentiation are fast, robust and cost-efficient [7]. They allow the simultaneous detection of the most relevant mammalian and poultry species for human consumption. Depending on sample matrix and processing grade, the limit of detection (LOD) ranges from 0.1% to 1%. A disadvantage of DNA arrays is that they do not yield quantitative information.

This limitation can be overcome by performing real-time PCR. However, quantification of animal species in meat products by real-time PCR is known to be a challenging task [1,3]. The main problem is to evaluate the meat content (*w*/*w*) one is actually interested in from the DNA concentration (e.g., ng/µL) determined by real-time PCR. Differences in tissue type, the number of cells per unit of mass, genome size, processing grade, and DNA extractability may impair the accuracy of quantitative results [8]. Various strategies have been proposed to compensate for these differences, e.g., the use of matrix-specific calibrators [9,10,11]. However, this strategy is very labor and time consuming. Thus, normalization with DNA extracts from material of defined composition [12] and relative quantification by using a reference real-time PCR assay [13,14,15] are widely applied in food control laboratories. With both approaches, the DNA ratios of the respective animal species in samples are obtained. Multiplex real-time PCR assays allow the identification of multiple species simultaneously, e.g., cattle, pig, turkey and chicken [16]; cattle, pig, equids and sheep [11]; roe deer, red deer, fallow deer and sika deer [17]; chicken, guinea fowl and pheasant or quail and turkey [18]. However, the number of species that can be targeted simultaneously is limited by the number of optical channels of the real-time PCR instrument.

In recent years, remarkable progress has been made towards developing DNA barcoding and DNA metabarcoding methods for food authentication [19,20,21,22,23]. DNA barcoding is based on amplification of short DNA barcode regions, followed by either high resolution melting (HRM) analysis [24,25] or Sanger sequencing [26,27]. DNA metabarcoding is the processing of multiple DNA templates using next-generation sequencing (NGS) technologies. While DNA barcoding via Sanger sequencing can only be applied for single species products, DNA metabarcoding also enables the identification of species in complex food and feed products containing multiple species. After amplifying the DNA barcode region, all amplicons, even those obtained for different samples, are sequenced in parallel. Finally, reads are analyzed using a bioinformatic workflow and compared to DNA reference sequences from well-characterized species for taxonomic assignment.

We have recently developed a DNA metabarcoding method allowing the identification of 15 mammalian and six poultry species [28]. The applicability of the method targeting a region of 16S rDNA was investigated by analyzing DNA extract mixtures and model sausages. The species of interest could be identified, differentiated and detected down to a proportion of 0.1%.

In the present study, we aimed at investigating the applicability of the DNA metabarcoding method for routine analysis in more detail. The design parameters and objectives of our study were as follows:

- The study included 25 reference samples with known composition, 56 commercial food and 23 pet food products.
- All samples were analyzed by the DNA metabarcoding method published previously [28] as well as by a commercial DNA array and/or by real-time PCR.
- Qualitative and quantitative results obtained by DNA metabarcoding were compared to those obtained by the two PCR methodologies currently playing the most important role in meat species authentication in official food laboratories.
- A subset of seven reference samples was analyzed by using the DNA metabarcoding method in two independent laboratories, yielding information on the robustness and reproducibility of the method.
- We evaluated whether the results obtained by DNA metabarcoding were in line with sample composition (reference samples) or declaration (commercial food and pet food products).

## 2. Materials and Methods

### 2.1. Samples

For this study, a collection of various samples was compiled. Reference samples, comprising eight meat mixtures (LGC7240-49), four dairy products (DLA45 1–4) and 13 boiled sausages (DLA44, DLAptAUS2, Lippold A–C 2019–2021), were supplied by regulatory authorities (LGC Standards Ltd., Teddington, UK; DLA—Proficiency Tests GmbH, Sievershütten, Germany; LVU Lippold, Herbolzheim, Germany). Food and pet food products were obtained from official food control agencies and supermarkets. The study mainly focused on sausages and pet food containing game species because these products are known to be vulnerable to the substitution of high-value game ingredients by lower-quality, cheaper meat species.

Reference samples were analyzed in “laboratory 1” (Chemical and Veterinary Analytical Institute Muensterland-Emscher-Lippe (CVUA-MEL) in cooperation with Chemical and Veterinary Analytical Institute Ostwestfalen-Lippe (CVUA-OWL), where sequencing was performed. A subset of seven reference samples was also analyzed in “laboratory 2” (Austrian Agency for Health and Food Safety (AGES)). Commercial food and pet food samples were analyzed independently either in laboratory 1 or laboratory 2.

### 2.2. DNA Extraction and Quantification

After homogenization and prior to DNA isolation, all samples were lysed in the presence of a lysis buffer and proteinase K solution at elevated temperature under constant shaking. Afterwards, DNA extraction was performed using commercially available kits. DNA from reference samples was isolated with either the Wizard Genomic DNA Purification Kit, the Wizard DNA Clean-Up Kit or the Maxwell 16 FFS Nucleic Acid Extraction Kit from Promega (Madison, WI, USA) according to the respective manufacturer’s instruction sheet. DNA from food and pet food samples was extracted with either the DNeasy mericon Food Kit (Qiagen, Hilden, Germany) or the Maxwell 16 FFS Nucleic Acid Extraction Kit (Promega, Madison, WI, USA), following the instructions of the manufacturers. DNA isolates were stored at −20 °C. Before DNA library preparation, the concentration of individual DNA extracts was determined either with a spectrophotometer (Eppendorf, Hamburg, Germany) or a Qubit 2.0 fluorometer (Thermo Fisher Scientific, Waltham, MA, USA) by using the dsDNA BR assay kit (Thermo Fisher Scientific, Waltham, MA, USA).

### 2.3. DNA-Library Preparation and NGS

A ~120 base pair fragment of the mitochondrial 16S rDNA gene was used as barcode region for species identification. Library preparation was carried out as described previously [28] with minor modifications. PCR products were indexed using the Illumina Nextera XT Index Kit v2 set A-D or the IDT-Illumina Nextera DNA UD Indexes Kit (Illumina, San Diego, CA, USA). Paired-end sequencing (2 × 150 bp) was performed with either the MiSeq Reagent Kit v2 or the MiSeq Reagent Kit v2 Micro (Illumina, San Diego, CA, USA) at a final loading concentration between 8–10 pM, depending on the instrument and the reagent kit, using the MiSeq system. PhiX DNA, added at a concentration of ~5%, served as sequencing control.

### 2.4. NGS Data Analysis Using Galaxy

After paired-end sequencing and FastQ file generation via on-board MiSeq Control software (version 2.6.2.1, Illumina, San Diego, CA, USA) and MiSeq Reporter software (version 2.6.2.3, Illumina, San Diego, CA, USA), the resulting FastQ files were used as input for data analysis. Afterwards, the previously uploaded files were processed according to the analysis pipeline as described previously [28] by using the Galaxy platform with the following modifications: the target-specific primer sequences were trimmed off with Cutadapt, Galaxy Version 1.16.6 [29] instead of using the tool Trim (Galaxy Version 0.0.1). Moreover, NGS reads were not clustered into operational taxonomic units (OTUs). After completely identical reads were collapsed into a representative sequence with the tool Dereplicate, Galaxy Version 1.0.0 [30], these sequences were directly matched against a customized database including 51 mitochondrial genomes from animals using BLASTn.

### 2.5. DNA Array and Real-Time PCR Assays

The LCD Array Kit MEAT 5.0 (Chipron GmbH, Berlin, Germany), allowing the simultaneous detection of 17 mammalian and seven bird species, was performed following the manufacturer’s instruction. Data analysis was done with the SlideReader Software (version 12, 2012-01, Chipron GmbH, Berlin, Germany).

Real-time PCR assays for the detection and quantification of meat species were performed following protocols published previously [11,14,16,18,31,32,33,34,35]. Quantification was carried out either by normalization with DNA extract from material of defined composition or relatively by using a reference real-time PCR assay [13].

## 3. Results and Discussion

In order to investigate the applicability of the DNA metabarcoding method for routine analysis, a total of 104 samples were analyzed. The samples consisted of 25 reference samples, 56 food products, and 23 pet food products. In addition to DNA metabarcoding, each sample was analyzed by real-time PCR and/or a commercial DNA array to evaluate the reliability of the DNA metabarcoding method. Results obtained by DNA metabarcoding are expressed as the ratio of the number of reads that were assigned to the respective meat species and the total number of reads that passed the amplicon analysis pipeline. The results obtained by the commercial DNA array are given as “positive” or “negative”, results obtained by real-time PCR as a ratio of DNA (%).

### 3.1. Reference Samples

Twenty-five reference samples were analyzed, comprising eight meat mixtures, four dairy products and thirteen boiled sausages. Reference samples contained from two to 14 meat species in a ratio from 1.0 to 99.0% (*w*/*w*) (Table 1). In total, 20 different animal species, including 14 mammalian species (moose, kangaroo, sheep, buffalo, horse, cattle, hare, goat, red deer, pork, rabbit, roe deer, reindeer and fallow deer) and six poultry species (ostrich, pheasant, Muscovy duck, turkey, goose, and chicken) were present in the reference samples. Results obtained by DNA metabarcoding, DNA array and real-time PCR assays are summarized in Table 1.

#### 3.1.1. Qualitative Results

The DNA metabarcoding method allowed the detection of 19 out of the 20 animal species covered by the reference samples. Fallow deer could not be detected because the DNA barcode region of fallow deer is not amplified due to two mismatches in the reverse primer (unpublished data). The DNA metabarcoding method allowed accurate identification of animal species in meat mixtures, dairy products, and boiled sausages. Species could be identified correctly down to a ratio of 1% (*w*/*w*). Goat DNA was detected at low concentration (0.3%) in one dairy sample (DLA45-2), although goat was not added intentionally. Notably, for this sample, proficiency test results were inconsistent (some were positive, some negative) [36].

The commercial DNA array and real-time PCR assays also allowed correct identification of all species contained. In contrast to the DNA metabarcoding method, goat was not detected in the dairy sample DLA45-2.

A subset of seven reference samples, including four dairy products (DLA45 1–4) and three boiled sausages (Lippold A–C, 2019), was independently subjected to DNA metabarcoding analysis at the AGES (laboratory 2, Table 1). In spite of small differences in the workflow, including a different sequencing chemistry, the species identified were identical, demonstrating the robustness of the DNA metabarcoding method. In line with laboratory 1, goat DNA was detected in dairy sample DLA45-2.

#### 3.1.2. Quantitative Results

**Figure 1:**
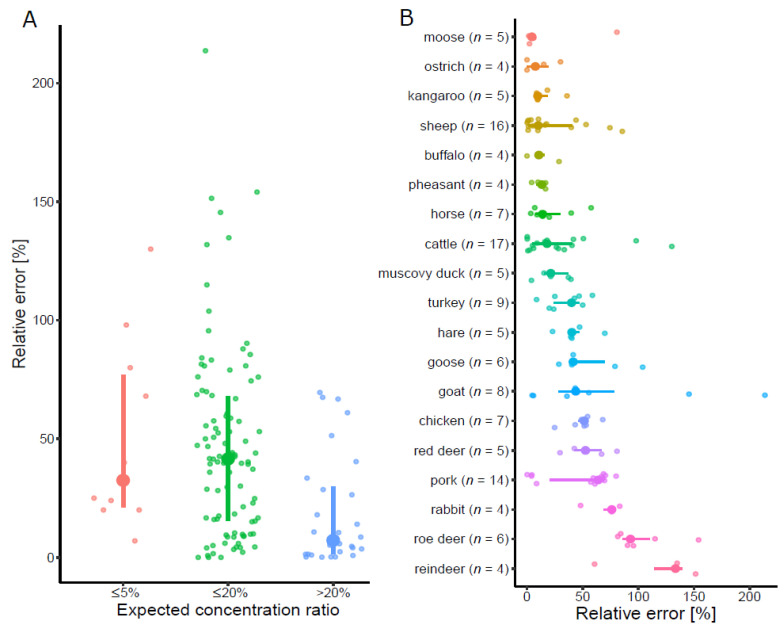
Relative quantification error (RQE) of the DNA-metabarcoding method on reference samples. RQE was calculated as the difference between the expected concentration ratio of a species and the proportion of reads assigned to that species, normalized by the expected concentration ratio. (**A**) RQE for different concentration ratio ranges. Small points represent a single measurement, large points and lines represent the median and inter-quantile range, respectively. Red: expected concentration <5%, green: expected concentration between 5% and 20%, blue: expected concentration >20%. (**B**) RQE by species. RQE calculated as for (**A**) is represented for each species, the number of data points (including those obtained in laboratory 2 (AGES)) is indicated in parenthesis. Species are sorted according to their median RQE from top (lowest) to bottom (highest). Small points represent a single measurement, large points and lines represent the median and inter-quantile range, respectively.

In order to investigate the applicability of the DNA metabarcoding method for obtaining quantitative results, we calculated the relative quantification error (RQE, absolute difference between the expected and experimentally determined ratio of the species contained in the sample, normalized by the expected value). RQE of the DNA metabarcoding method depended on the ratio of the species in the reference sample (Figure 1A). For species being present at a concentration ratio ≤5%, the median of RQE was 33%. For concentration ratios ranging from 5% to 20%, the median RQE was slightly higher (42%). As expected, the lowest RQE (7%) was obtained for concentration ratios >20%.

In Figure 1B, the RQE is shown for each of the 19 species detected by DNA metabarcoding. For eight mammalian (moose, kangaroo, sheep, buffalo, horse, cattle, hare, and goat) and five poultry species (ostrich, pheasant, Muscovy duck, turkey, and goose), the median RQE was <50%. For four mammalian species (red deer, pork, rabbit, and roe deer) and chicken, the median RQE was between 50% and 100%. The highest median RQE was obtained for reindeer (133%).

**Figure 2:**
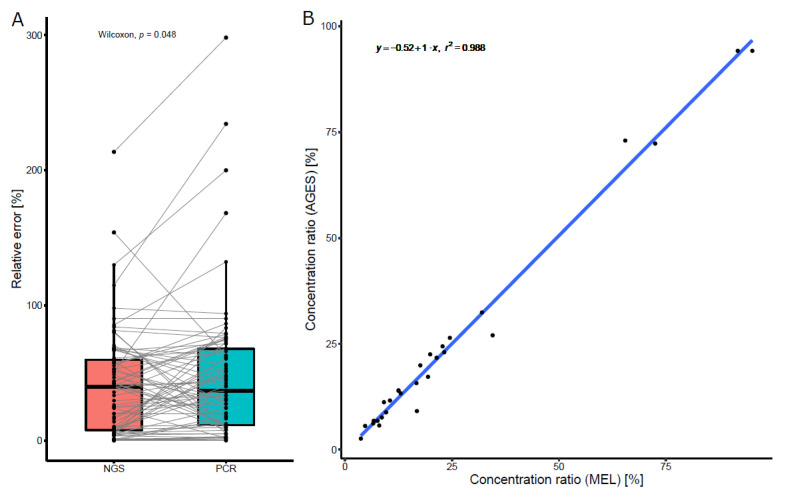
Precision and reproducibility of the DNA-metabarcoding method. (**A**) Comparison of RQE of the DNA metabarcoding method (red) compared to that of real-time PCR (blue). Only species for which quantitative PCR was performed are represented. Black points represent single measurement and grey lines connect paired values. Colored boxes represent the interquartile range with the horizontal line at the median and whiskers represent the Tukey-corrected minimum and maximum. Although a significant difference between the two distributions was calculated (paired Wilcoxon rank test *p* = 0.048), the quantitative difference is too small to be biologically relevant. (**B**) Reproducibility of DNA metabarcoding quantification in two different laboratories. A subset of the samples was quantified with the DNA metabarcoding method in laboratory 1 (CVUA-MEL, x-axis) and laboratory 2 (AGES, y-axis), with highly similar results. A linear regression (blue) of both datasets showed a slope of 1 and a Pearson correlation coefficient r² = 0.988. Each point represents a single observation.

RQE was also calculated for real-time PCR (difference between the ratio of the species contained in the reference sample (Table 1, column 3) and the ratio of DNA (%) determined by real-time PCR (Table 1, column 5), divided by the ratio of the species contained in the reference sample (Table 1, column 3)). The boxplot in Figure 2A shows the distributions of RQE determined by DNA metabarcoding and real-time PCR. Median and interquartile ranges for NGS and PCR errors are 39.7% (7.8%–59.9%) and 36.9% (11.4%–67.9%), respectively, indicating that the two distributions largely overlap.

For all major components (cattle, sheep; 95% or 99%) in meat mixtures, the RQE of the DNA barcoding method and real-time PCR was <6%. For the minor component (horse, turkey; 1%, 5%) in samples LGC7240, LGC7247, and LGC7246, the RQE of both methods was <30%. Both DNA metabarcoding and real-time PCR led to substantially too high ratios (RQE 94%—200%) for cattle as minor component (LGC7249, 5%; LGC7248, 1%). The content of pork (1%) in sample LGC7242 was substantially overestimated (RQE 80%) by DNA metabarcoding, but not by real-time PCR.

Each of the four dairy products contained one major component (cattle, buffalo, or goat) and one, two, or three minor components (buffalo, cattle, sheep, or goat). The major components could be quantified with the RQE <30% with both methods. Only in sample DLA45-3, cattle was substantially underestimated by real-time PCR (RQE 37%). Due to high lipid content and harsh processing procedures, DNA isolated from dairy products is frequently not amplified efficiently [37]. Underestimation of cow milk compared to goat milk by real-time PCR has already been reported by Rentsch et al. and was explained by the relatively low number of somatic cell counts in cow milk compared to goat milk [31]. In the case of minor components, for buffalo (8%) and cattle (10%) in samples DLA45-1 and DLA45-2, respectively, the RQE of DNA metabarcoding and real-time PCR was ≤24%. Goat (11%) was substantially overestimated in sample DLA45-3 (RQE 214% and 298%), and sheep (10%) substantially underestimated in DLA45-4 (RQE 53% and 66%) by DNA barcoding and real-time PCR.

The number of species in 13 boiled sausages ranged from two (DLA44-1) to 14 (Lippold-C, 2020 and Lippold-A, 2021). For major components at a ratio >85% (pork in samples DLA44-1, DLA44-3, and DLAptAUS2-3.1), the RQE of DNA metabarcoding and real-time PCR was <10%. The major components at a ratio of between 85% and 20% (Lippold-A, 2013: cattle, chicken; Lippold-C, 2019: pork; Lippold-A, 2020: horse; Lippold-B, 2020: pork; Lippold-B, 2021: pork; Lippold-C, 2021: cattle) were underestimated by DNA metabarcoding and real-time PCR, with the RQE ranging from 33% to 67% and 31% to 75%, respectively. A number of minor components at a ratio of between 20% and 5% could be quantified with RQE <30% by either DNA metabarcoding (e.g., Lippold-A, 2013: sheep, Muscovy duck; Lippold-A, 2019; Lippold-C, 2020: sheep), or real-time PCR (e.g., Lippold-A, 2019: red deer; Lippold-B, 2019: chicken, turkey) or both methods (e.g., Lippold-A, 2019: sheep, pheasant; Lippold-B, 2020: chicken).

For cattle in samples Lippold-C, 2019 and Lippold-B, 2021 ratios of 1.1/1.2% (NGS) and 1.8% (PCR) or 2.8% (NGS) and 1.8% (PCR) were determined, respectively. Cattle was not added intentionally to these samples, but was contained as traces probably due to production-related carryover. Results of both proficiency tests showed that most participants (86% and 97%) also identified cattle in these samples.

Quantitative data sets obtained for the subset of seven reference samples analyzed in laboratory 1 and laboratory 2 by DNA metabarcoding showed a very good correlation (r^2^ = 0.988) (Figure 2B), indicating the high reproducibility of the method. In conclusion, we found that the RQE was quite variable and depended on both the concentration and the identity of the analyte. Additionally, the error was comparable to that of PCR, the current gold-standard method.

Overall our data confirm the limitations known for DNA quantification in meat products [23]. Due to the differences in tissue type, the number of cells per unit of mass, genome size, processing grade and DNA extractability, quantitative results derived from DNA-based methods should serve only as rough estimates for weight ratios of different species in food and feed [8]. During manual and industrial production of meat products production-related carryover of undeclared animal species regularly occurs. In routine analysis of samples in public laboratories, mass concentrations below 1% (*w*/*w*) are generally reported as possible process contaminants and do not constitute a violation of declaration. Considering the high quantitation errors of DNA-based methods, in most cases a factor of five might be appropriate to discriminate between production-related carryover of undeclared species and mislabeling.

### 3.2. Commercial Food Products

Table 2 summarizes the results obtained by DNA metabarcoding, real-time PCR and DNA array for 56 commercial food products obtained from food control agencies or purchased at local supermarkets. The samples comprised 34 sausages, including seven wild boar sausages, 20 deer sausages and seven further sausages, six vertical rotating meat spits, seven pâtés, two minced meat products, one steak, two convenience foods, and four milk products.

Table 2 indicates that DNA metabarcoding and real-time PCR and/or the commercial DNA array led to identical qualitative results for the 56 commercial food products. However, for discrimination of meat from wild boar (*Sus scrofa scrofa*) and meat from domestic pig (*Sus scrofa domesticus*), results of two singleplex real-time PCR assays and/or a duplex real-time PCR assay developed recently had to be taken into account [38]. Neither the DNA metabarcoding method nor common real-time PCR assays for pork allow distinguishing between wild boar and pork, yielding only information on the total ratio of wild boar and pork DNA. This is due to the fact that the genomes of the two subspecies are highly homologous and hybridization and back-crossings increased sequence homologies and intra-subspecies variability [39,40].

The ingredient list of 14 out of 20 deer sausages did not contain any information on the deer species (red deer, sika deer, fallow deer). Red deer, roe deer, red deer and roe deer, and red deer and sika deer were detected with DNA ratios >1% in eight, one, three and two of these sausages, respectively. Four and two out of the 20 deer sausages were declared to contain roe deer and red deer, respectively. Our results confirmed the presence of these deer species in the respective food products.

For all species detected in deer sausage 17, sausage 5 and 6, pâté 7 and minced meat product 1 (Table 2), the ratios obtained by DNA metabarcoding and real-time PCR differed by less than 30%. However, in the cases of the other food products, differences >30% were observed for at least one of the species identified.

**Figure 3:**
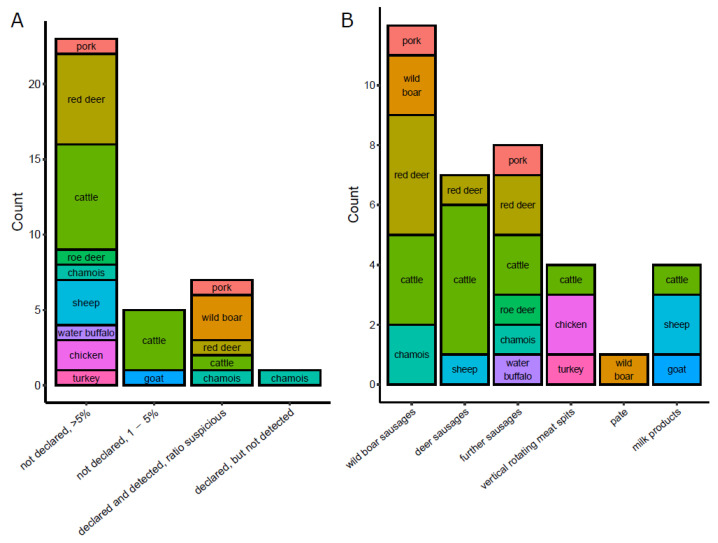
Wrong declarations in foodstuffs (**A**) Breakdown of wrongly labeled species by type of fraud in foodstuffs. Each box represents a single species, the size of the box indicates the number of times that this species appeared for each type of fraud in the dataset. (**B**) Breakdown of wrongly labelled species by type of food product. Each box represents a single species, the size of the box indicates the number of times that this species appeared for each type of food product in the dataset.

Comparison of our results, obtained by DNA metabarcoding and real-time PCR and/or the DNA array, with the food ingredient lists revealed multiple discrepancies (Table 2). In a number of commercial food products, species that were not given on the food label were detected by both DNA metabarcoding and real-time PCR and/or the DNA array. Most frequently, the DNA of undeclared species was found in high ratios >5%, indicating that the replacement of meat species by cheaper alternatives is an ongoing food fraud issue. For some products, the species detected were declared but the DNA ratios determined did not correspond with declaration (“declared and detected, ratio suspicious”). In further products, the DNA of undeclared species was detected in traces between 1% and 5%, which were possibly contained due to production-related carry-over. In only one product (wild boar sausage 5), a species declared (chamois) was not detected. Figure 3A summarizes the number of mislabeled species by type of fraud in commercial foodstuffs, Figure 3B the number of mislabeled species by type of food product.

### 3.3. Commercial Pet Food Products

The applicability of the DNA metabarcoding method was also investigated by analyzing 23 pet food products. The following species were given on the food label: deer, roe deer, cattle, sheep, rabbit, chicken, turkey, duck, Muscovy duck, and ostrich. Table 3 indicates that qualitative results obtained by DNA metabarcoding were in line with those obtained by real-time PCR and/or the commercial DNA array. For some animal species, e.g., red deer in samples 1, 3, 12; pork in sample 2, 10, 19, 21; and chicken in samples 19, 22; the ratios determined by DNA metabarcoding and real-time PCR differed by less than 30%. However, in other cases, differences in the ratios >30% were obtained (Table 3).

Fifteen out of the 23 pet food products were declared to contain deer, without disclosing the deer species. DNA metabarcoding and real-time PCR and/or the commercial DNA array detected red deer in six, red deer and roe deer in four and reindeer in one out of these 15 pet food products. In four pet food products (samples 5, 8, 11, and 21), deer was neither detected by DNA metabarcoding nor by real-time PCR and/or the commercial DNA array. Identical qualitative results were also obtained for three pet food products declared to contain roe deer (samples 12, 16, and 18). Each of the methodologies applied yielded a negative result for roe deer, but a positive result for red deer.

In sample 18, sika deer (16.6%) was detected by DNA metabarcoding. Since sika deer is rarely used in pet food products, sample 18 was not analyzed by a real-time PCR assay for sika deer and the DNA array used does not detect sika deer. This example illustrates one of the main limitations of using PCR for meat species authentication: animal species that are not expected will not be detected [41].

**Figure 4:**
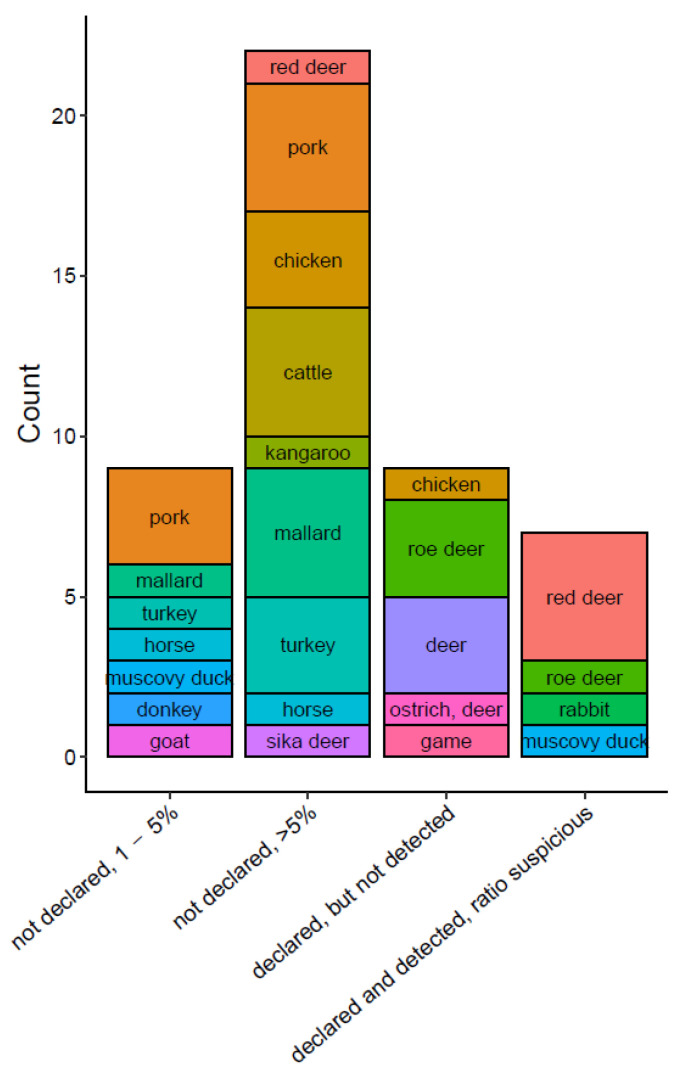
Breakdown of wrongly labelled species by type of fraud in pet food products. Each box represents a single species, the size of the box indicated the number of times that this species appeared for each type of fraud in the dataset.

In a high number of commercial pet food products, undeclared species were detected by each of the methodologies applied. Most frequently, undeclared species, were present at a ratio >5%, e.g., pork, chicken, cattle, mallard, and turkey (Figure 4). These animal species of lower commercial value mainly replaced deer, either totally or in part. The results show that inspection of pet food for authenticity has high relevance.

In some products, undeclared species were detected in a ratio between 1% and 5%. Most probably, these species were present due to production-related carry-over. Chicken, roe deer, deer, ostrich and game could not be identified in several pet food products although they were declared to contain these species. In some products, the declared species was detected but the DNA ratio determined drastically differed from the content given on the label (Figure 4). These results are probably caused by total or partial degradation of DNA due to high processing grades of the respective raw materials.

### 3.4. Cost Analysis

**Figure 5:**
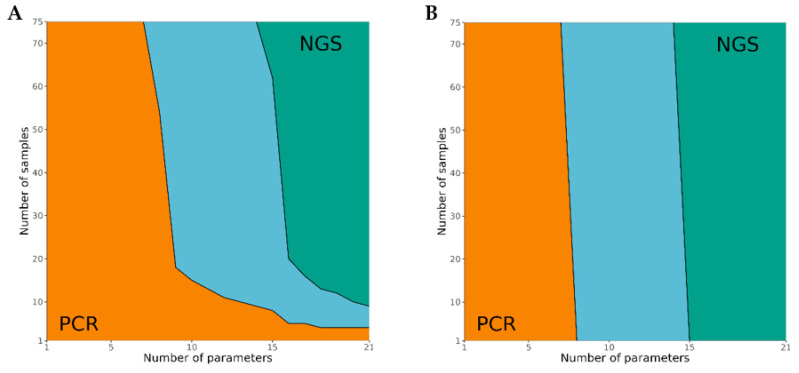
Break-even point analysis of NGS-metabarcoding and real-time PCR for the qualitative identification of bird and mammal species. The left-most orange area corresponds to combinations of sample number/parameter number for which PCR is always cheaper than NGS. The right-most green area corresponds to combinations for which NGS is always cheaper than PCR. The middle blue zone corresponds to combinations for which the cost difference largely depends on the degree of multiplexing of the PCRs. NGS costs were calculated for two exemplary laboratories: (**A**) a laboratory running exclusively meat-metabarcoding runs, and (**B**) a laboratory running full-capacity sequencing runs, for example, mixing samples with other type of assays.

Metabarcoding could be an attractive alternative to real-time PCR in species differentiation, especially due to the possibility of analyzing many samples simultaneously for many species. A detailed cost comparison with the standard real-time PCR method is not yet available. For the present publication a break-even analysis was performed, based on current data from AGES cost accounting, to show what effect the number of samples and the number of parameters (animal species) has on the choice of methodology used. The break-even point or volume (BEP) represents the number of tested samples/parameters where the real-time PCR-based cost equals the NGS-based cost. Above this threshold, an NGS-based approach generates savings. Figure 5A shows the BEP for NGS of a maximum of 21 animal species, corresponding to 21 real-time PCR methods for animal species available in the AGES laboratory. The analysis shows that the use of NGS is more cost-effective for the detection of 21 animal species from the tenth sample onwards. If no multiplex methods for real-time PCR are available in the laboratory, NGS is already profitable from the fifth sample onwards. If the scope of testing is limited to only up to seven animal species per sample, real-time PCR is always cheaper than NGS analysis. Figure 5B shows the BEP at full capacity of the sequencing kit. If the sequencing kit is fully utilized (Illumina MiSeq v2 chemistry, 75 samples, 200,000 reads per sample), the costs per sample are significantly reduced. In this case, NGS is already cheaper from the first sample onwards, if at least 15 parameters are analyzed. Below a parameter number of seven, however, real-time PCR always remains the cheaper method.

## 4. Conclusions

By analyzing 25 reference samples, 56 commercial food and 23 pet food products using DNA metabarcoding and real-time PCR and/or a commercial DNA array, we demonstrated that the DNA metabarcoding method developed recently is a suitable screening method for meat species authentication. Qualitative and quantitative results of the DNA metabarcoding method were in line with those obtained by real-time PCR. The results from independent analyses in two laboratories indicate the robustness and reproducibility of the DNA metabarcoding method. Our data on reference samples confirm the limitations known for DNA quantification in meat products. Quantitative results derived from DNA-based methods should serve only as rough estimates for weight ratios of different species in food and feed.

A major advantage of metabarcoding is the parallel detection of a large number of animal species including species not tested routinely or for which no real-time PCR methods are available. Our results indicate that in addition to food products, DNA metabarcoding is particularly applicable to pet food products, which frequently contain multiple animal species and were shown to be also highly prone to adulteration.

For a large number of samples or parameters, metabarcoding is the more cost-effective analysis. By combining different applications (joint sequencing of plant and animal species, bacteria, etc.), an additional cost reduction is possible, as the sequencing kits, the biggest cost driver, can be better utilized.

## Figures and Tables

**Table 1:** Results obtained for reference samples. DNA array and real-time PCR results were obtained in laboratory 1. DNA metabarcoding results were obtained in laboratory 1, except those marked by footnote 5.

Reference Sample	Composition		Results	
	Species	Ratio (%, *w*/*w*)	DNA Metabarcoding Ratio of Reads (%) ^4^	Real-Time PCR (Ratio of DNA (%)) or DNA Array (Positive/Negative)
LGC7242	cattle	99.0	98.2	98.9 ^1^
	pork	1.0	1.8	1.1 ^1^
LGC7240	cattle	99.0	98.8	95.9 ^1^
	horse	1.0	1.2	1.3 (Equidae) ^1^
LGC7249	sheep	95.0	90.1	90.3 ^1^
	cattle	5.0	9.9	9.7 ^1^
LGC7248	sheep	99.0	97.7	97.0 ^1^
	cattle	1.0	2.3	3.0 ^1^
LGC7245	sheep	95.0	98.4	93.0 ^1^
	chicken	5.0	1.6	7.0 ^1^
LGC7244	sheep	99.0	100.0	99.9 ^1^
	chicken	1.0	<0.1	0.1 ^1^
LGC7247	sheep	95.0	96.3	94.9 ^1^
	turkey	5.0	3.8	5.1 ^1^
LGC7246	sheep	99.0	98.8	98.8 ^1^
	turkey	1.0	1.2	1.2 ^1^
DLA44-1, 2019	pork	93.4	89.6	88.5 ^1^
	horse	6.6	10.4	11.5 (Equidae) ^1^
DLA44-3, 2019	pork	87.3	87.4	85.1 ^1^
	turkey	7.0	7.6	11.3 ^1^
	cattle	5.6	5.1	3.6 ^1^
DLA45-1, 2019	cattle	92.0	91.8/94.2 ^5^	90.7 ^1^
	buffalo	8.0	8.0/5.7 ^5^	9.3 ^1^
DLA45-2, 2019	buffalo	81.0	72.5/72.3 ^5^	71.5 ^1^
	cattle	10.0	10.5/11.6 ^5^	7.6 ^1^
	sheep	9.0	16.7/15.7 ^5^	20.9 ^1^
	goat	not added ^6^	0.3/0.3 ^5^	negative ^3^
DLA45-3, 2019	cattle	89.0	65.5 / 73.0 ^5^	56.2 ^1^
	goat	11.0	34.5/27.0 ^5^	43.8 ^1^
DLA45-4, 2019	goat	90.0	95.2/94.2 ^5^	96.9 ^1^
	sheep	10.0	4.7/5.6 ^5^	3.4 ^1^
DLAptAUS2-3.1, 2020	pork	90.9	98.7	99.7 ^1^
	donkey	9.1	1.1	positive ^3^
	horse	not added ^6^	0.2	0.3 (Equidae) ^1^
Lippold-A, 2013	cattle	27.8	18.5	14.7 ^2^
	sheep	16.7	14.0	6.6 ^2^
	chicken	22.2	10.8	15.3 ^2^
	goose	11.1	15.7	positive ^3^
	Muscovy duck	11.1	12.8	positive ^3^
	roe deer	11.1	28.2	18.1 ^2^
Lippold-A, 2019	red deer	16.0	22.8/24.4 ^5^	13.2 ^2^
	cattle	15.6	9.1/11.2 ^5^	22.2 ^2^
	ostrich	15.3	17.6/19.9 ^5^	positive ^3^
	hare	14.4	8.6/7.6 ^5^	positive ^3^
	kangaroo	14.2	16.8/9.1 ^5^	positive ^3^
	sheep	12.6	12.5/13.9 ^5^	10.3 ^2^
	pheasant	12.0	12.5/14.0 ^5^	10.5 ^2^
Lippold-B, 2019	goose	16.4	23.2/23.0 ^5^	positive ^3^
	rabbit	15.5	3.7/2.6 ^5^	positive ^3^
	chicken	14.9	7.6/6.8 ^5^	16.6 ^2^
	pork	13.6	21.4/21.7 ^5^	2.9 ^2^
	moose	13.6	13.0/13.3 ^5^	positive ^3^
	roe deer	13.5	24.5/26.4 ^5^	23.8 ^2^
	turkey	12.4	6.6/6.2 ^5^	8.7 ^2^
Lippold-C, 2019	pork	28.9	9.6/8.8 ^5^	8.2 ^2^
	horse	17.8	19.4/17.2 ^5^	10.6 (Equidae) ^2^
	Muscovy duck	16.4	19.9/22.5 ^5^	positive ^3^
	reindeer	13.8	32.0/32.4 ^5^	positive ^3^
	goat	12.0	6.7/6.8 ^5^	2.8 ^2^
	fallow deer	11.1	-	12.6 ^2^
	cattle	traces ^7^	1.1/1.2 ^5^	1.8 ^2^
Lippold-A, 2020	goose	38.8	49.9	positive ^3^
	horse	25.0	28.5	12.9 (Equidae) ^2^
	pork	12.5	3.7	9.1 ^2^
	hare	11.2	6.8	positive ^3^
	Muscovy duck	10.0	9.6	positive ^3^
	turkey	2.5	1.5	2.3 ^2^
Lippold-B, 2020	pork	31.3	12.2	10.2 ^2^
	fallow deer	24.1	-	12.9 ^2^
	reindeer	17.9	45.0	positive ^3^
	chicken	12.5	9.4	15.9 ^2^
	goat	11.7	7.5	3.7 ^2^
	turkey	2.4	1.8	1.6 ^2^
Lippold-C, 2020	goose	8.1	14.5	positive ^3^
	red deer	8.1	10.5	10.8 ^2^
	cattle	7.9	3.9	21.2 ^2^
	rabbit	7.7	4.0	positive ^3^
	chicken	7.4	4.2	13.0 ^2^
	hare	7.3	2.2	positive ^3^
	kangaroo	7.2	6.5	positive ^3^
	pork	6.7	11.3	2.5 ^2^
	moose	6.7	7.1	positive ^3^
	roe deer	6.7	14.4	22.4 ^2^
	sheep	6.3	5.2	2.8 ^2^
	turkey	6.1	3.5	5.4 ^2^
	pheasant	6.0	5.0	positive ^3^
	ostrich	7.7	7.7	positive ^3^
Lippold-A, 2021	cattle	8.5	8.0	4.1 ^2^
	pork	6.3	10.6	3.1 ^2^
	sheep	7.8	4.7	6.2 ^2^
	horse	6.3	3.8	3.5 (Equidae) ^2^
	red deer	7.8	14.1	7.4 ^2^
	fallow deer	6.3	-	3.8 ^2^
	roe deer	6.3	11.6	11.3 ^2^
	moose	6.3	6.4	positive ^3^
	kangaroo	7.4	8.1	positive ^3^
	rabbit	7.1	1.7	positive ^3^
	reindeer	6.1	9.8	positive ^3^
	chicken	9.8	4.6	12.2 ^2^
	turkey	6.3	2.6	5.7 ^2^
	ostrich	7.8	7.8	positive ^3^
Lippold-B, 2021	cattle	traces ^7^	2.8	1.8 ^2^
	pork	32.6	10.6	14.2 ^2^
	horse	4.3	4.0	2.0 (Equidae) ^2^
	roe deer	14.4	27.4	27.4 ^2^
	moose	10.9	19.7	positive ^3^
	kangaroo	13.9	12.7	positive ^3^
	hare	10.9	8.4	positive ^3^
	pheasant	13.1	14.4	positive ^3^
Lippold-C, 2021	cattle	25.0	14.9	6.2 ^2^
	pork	13.9	14.5	2.3 ^2^
	sheep	14.3	12.9	3.9 ^2^
	goat	16.4	7.3	2.2 ^2^
	red deer	12.1	20.2	6.6 ^2^
	goose	7.8	15.9	positive ^3^
	Muscovy duck	10.4	14.5	positive ^3^

-: Not detected. ^1^ Relative quantification based on normalization. ^2^ Relative quantification by using a reference real-time PCR assay. ^3^ Obtained by the DNA array. ^4^ For samples containing fallow deer, ratios of reads refer to 100% minus ratio (%, *w*/*w*) of fallow deer. ^5^ Obtained in laboratory 2 (AGES). ^6^ Proficiency test results were inconsistent, some were positive, some negative. ^7^ Species not added intentionally, but identified by 86% (Lippold-C, 2019) and 97% (Lippold-B, 2021) of the participants of the proficiency test.

**Table 2:** Results for commercial food products.

		Result			
Sample	Animal Species Declared	Animal Species Detected	DNA Metabarcoding Ratio of Reads (%)	Real-Time PCR (Ratio of DNA (%)) or DNA Array (Positive/Negative)	Comment
wild boar sausage 1	wild boar, pork, pork bacon	pork	83.0 ^4^	52.5 ^1^	
		wild boar		35.2 ^1^	
		red deer	15.1	3.9 ^1^	not declared, >5%
		cattle	1.7	8.4 ^1^	not declared, 1%–5%
wild boar sausage 2	wild boar, pork, pork bacon	wild boar	86.9 ^4^	23.7 ^1^	
		pork		64.1 ^1^	
		red deer	13.1	12.3 ^1^	not declared, >5%
wild boar sausage 3	55% wild boar, 36% roe deer	roe deer	60.7	40.8 ^1^	
		pork	25.1 ^4^	50.5 ^1^	
		wild boar		<1.0 ^1^	declared and detected ^3^
		cattle	14.0	8.7 ^1^	not declared, >5%
wild boar sausage 4	74% red deer, 22% wild boar bacon	cattle	30.2	46.4 ^1^	not declared, >5%
		pork	28.8 ^4^	43.5 ^1^	not declared, >5%
		wild boar		<1.0 ^1^	declared and detected, r.s.
		red deer	22.8	10.1 ^1^	declared and detected, r.s.
		chamois	18.2	-	not declared, >5%
wild boar sausage 5	chamois, wild boar, roe deer, pork bacon	pork	48.8 ^4^	8.9 ^1^	
		wild boar		38.2 ^1^	
		red deer	36.8	42.0 ^1^	not declared, >5%
		roe deer	14.0	10.9 ^1^	
		chamois	0.0	-	declared, but not detected
wild boar sausage 6	no declaration	pork	62.2 ^4^	28.5 ^1^	
		wild boar		26.1 ^1^	
		roe deer	24.4	16.0 ^1^	
		cattle	13.4	29.4 ^1^	
wild boar sausage 7	game, cattle, pork bacon	pork	70.2 ^4^	60.3 ^1^	
		wild boar		16.0 ^1^	
		cattle	28.7	23.7 ^1^	
		roe deer	<1.0	<1.0 ^1^	
		sheep	<1.0	<1.0 ^1^	
deer sausage 1	deer, pork, pork bacon	red deer	72.0	52.6 ^1^	
		pork	19.5	41.6 ^1^	
		cattle	8.5	5.8 ^1^	not declared, >5%
deer sausage 2	roe deer, pork, pork bacon	roe deer	52.0	28.3 ^1^	
		pork	22.8 ^4^	54.3 ^1^	
		wild boar		<1.0	
		cattle	10.8	7.9 ^1^	not declared, >5%
		red deer	14.3	9.5 ^1^	not declared, >5%
deer sausage 3	roe deer, pork	roe deer	89.9	75.1 ^1^	
		pork	5.9	23.0 ^1^	
		cattle	4.2	1.9 ^1^	not declared, 1%–5%
deer sausage 4	deer, pork	red deer	67.0	52.3 ^1^	
		pork	33.0 ^4^	47.7 ^1^	
		wild boar		<1.0 ^1^	
deer sausage 5	roe deer, pork, pork bacon	roe deer	81.5	78.5 ^1^	
		pork	9.3	15.8 ^1^	
		cattle	9.0	5.7 ^1^	not declared, >5%
		red deer	< 1.0	<1.0 ^1^	
deer sausage 6	game, pork	red deer	83.8	66.9 ^1^	
		cattle	9.3	14.9 ^1^	not declared, >5%
		pork	5.2 ^4^	15.3 ^1^	
		wild boar		<1.0	
		roe deer	1.7	3.0 ^1^	
deer sausage 7	70% red deer, 30% pork	red deer	70.4	72.2 ^1^	
		pork	29.6 ^4^	<1.0 ^1^	
		wild boar		27.8 ^1^	
deer sausage 8	deer, pork, pork bacon	red deer	68.0	46.7 ^1^	
		pork	31.7	53.3 ^1^	
		sika deer	<1.0	-	
deer sausage 9	deer, pork, pork bacon	roe deer	79.0	58.7 ^1^	
		pork	20.9	41.3 ^1^	
deer sausage 10	deer, pork, pork bacon	red deer	74.1	38.5 ^1^	
		pork	25.3	61.5 ^1^	
deer sausage 11	deer, pork, pork bacon	red deer	72.0	36.4 ^1^	
		pork	27.8	63.6 ^1^	
deer sausage 12	pork, red deer	pork	66.6	51.9 ^2^	
		red deer	33.4	48.1 ^2^	
deer sausage 13	roe deer, pork, pork bacon	roe deer	81.6	67.4 ^1^	
		pork	18.3 ^4^	32.6 ^1^	
		wild boar		<1.0 ^1^	
deer sausage 14	deer, pork, pork bacon, cattle casing ^5^	red deer	70.6	48.7 ^1^	
		pork	25.7	50.0 ^1^	
		sika deer	2.6	1.3 ^1^	
		roe deer	<1.0	<1.0 ^1^	
deer sausage 15	pork, deer, cattle	red deer	51.4	39.9 ^1^	
		pork	31.1	45.3 ^1^	
		cattle	16.9	14.8 ^1^	
		roe deer	< 1.0	<1.0 ^1^	
deer sausage 16	deer, pork, pork bacon, cattle casing ^5^	red deer	53.6	27.2 ^1^	
		pork	24.2	61.3 ^1^	
		sheep	21.6	11.4 ^1^	not declared, >5%
		roe deer	<1.0	<1.0 ^1^	
		fallow deer	-	<1.0 ^1^	
deer sausage 17	deer, pork, cattle	roe deer	55.8	59.0 ^1^	
		red deer	24.5	24.6 ^1^	
		pork	10.2 ^4^	8.9 ^1^	
		wild boar		<1.0 ^1^	
		cattle	9.4	7.5 ^1^	
deer sausage 18	deer, cattle	red deer	66.1	53.8 ^1^	
		cattle	32.2	46.2 ^1^	
		sika deer	1.3	<1.0 ^1^	
deer sausage 19	deer, cattle	red deer	76.9	43.9 ^1^	
		cattle	20.1	56.1 ^1^	
		sika deer	2.9	<1.0 ^1^	
deer sausage 20	game, cattle, pork bacon	red deer	78.3	80.9 ^1^	
		roe deer	14.5	7.0 ^1^	
		pork	5.4	10.4 ^1^	
		cattle	1.8	1.7 ^1^	
sausage 1	chamois, cattle, pork bacon	red deer	35.8	11.9 ^1^	not declared, >5%
		pork	29.2	70.0 ^1^	declared and detected, r.s.
		cattle	19.8	8.9 ^1^	
		roe deer	13.4	9.2 ^1^	not declared, >5%
		chamois	1.6	-	declared and detected, r.s.
sausage 2	60% sheep, 35% pork, 5% goat	sheep	44.5	35.7 ^1^	
		pork	27.0	49.7 ^1^	
		red deer	12.5	3.9 ^1^	not declared, >5%
		cattle	8.5	9.4 ^1^	not declared, >5%
		goat	7.4	1.4 ^1^	
sausage 3	cattle	water buffalo	67.0	-	not declared, >5%
		cattle	33.0	22.9 ^2^	
sausage 4	42% cattle, 35% chicken	chicken	86.0	96.4 ^1^	
		cattle	13.5	3.6 ^1^	declared and detected, r.s.
sausage 5	40% poultry, 15% cattle, cattle fat	turkey	44.4	36.4 ^1^	
		chicken	30.1	32.9 ^1^	
		cattle	25.0	30.7 ^1^	
sausage 6	pork, cattle or lamb	cattle	53.6	59.5 ^1^	
		pork	46.1	40.5 ^1^	
sausage 7	lamb, pork	sheep	80.1	71.7 ^1^	
		pork	19.8	28.3 ^1^	
vertical rotating meat spit 1	95% beef	cattle	64.9	85.4 ^1^	
		turkey	35.1	14.6 ^1^	not declared, >5%
vertical rotating meat spit 2	75% veal, 20% turkey	cattle	57.5	76.0 ^1^	
		turkey	35.4	21.1 ^1^	
		chicken	7.1	2.9 ^1^	not declared, >5%
vertical rotating meat spit 3	70% veal, 20% turkey	cattle	59.2	74.1 ^1^	
		turkey	33.7	21.5 ^1^	
		chicken	7.2	4.4 ^1^	not declared, >5%
vertical rotating meat spit 4	turkey	turkey	98.2	94.8 ^1^	
		cattle	1.7	5.2 ^1^	not declared, 1%–5%
vertical rotating meat spit 5	55% cattle, 10% turkey, 25% chicken	cattle	58.8	29.1 ^1^	
		chicken	23.0	35.0 ^1^	
		turkey	18.1	35.9 ^1^	
vertical rotating meat spit 6	55% cattle, 35% poultry	cattle	56.0	41.9 ^1^	
		chicken	43.5	58.1 ^1^	
		turkey	< 1.0	<1.0 ^1^	
pâté 1	wild boar, pork	pork	99.8 ^4^	100.0 ^1^	
		wild boar		<1.0 ^1^	declared and detected, r.s.
pâté 2	game, pork	pork	57.6	77.5 ^1^	
		red deer	42.1	22.5 ^1^	
pâté 3	49% pork, lamb liver	pork	66.6	71.9 ^1^	
		sheep	33.4	28.1 ^1^	
pâté 4	pork neck and liver, rabbit meat	pork	96.2	46.5 ^2^	
		rabbit	3.8	positive ^3^	
pâté 5	duck meat and breast, poultry liver	turkey	49.1	21.3 ^2^	
		mallard	28.1	positive ^3^	
		Muscovy duck	22.8	positive ^3^	
pâté 6	50% pork meat, 20% red deer meat	pork	57.9	84.4 ^2^	
		red deer	42.0	15.6 ^2^	
pâté 7	33% pork meat, 20% roe deer meat	roe deer	59.7	59.9 ^2^	
		pork	40.3	40.1 ^2^	
minced meat product 1	chicken, cattle	chicken	76.2	81.9 ^2^	
		cattle	23.0	18.1 ^2^	
		buffalo, kangaroo, fish	<1.0	positive ^3^	
minced meat product 2	lamb, cattle	cattle	70.3	51.6 ^1^	
		sheep	29.4	48.4 ^1^	
steak	reindeer	reindeer	100.0	positive ^3^	
convenience food 1	37% pork and cattle, cattle soup	pork	67.9	82.0 ^1^	
		cattle	32.1	18.0 ^1^	
convenience food 2	25% pork, cattle soup	pork	87.2	93.9 ^1^	
		cattle	12.5	6.1 ^1^	
milk product 1	goat	goat	97.4	positive ^3^	
		cattle	2.6	positive ^3^	not declared, 1%–5%
milk product 2	goat milk	goat	62.8	positive ^3^	
		sheep	36.2	positive ^3^	not declared, >5%
		ibex	<1.0	-	
		cattle	<1.0	positive ^3^	
milk product 3	goat milk	goat	62.9	positive ^3^	
		sheep	36.0	positive ^3^	not declared, >5%
		ibex	<1.0	-	
		cattle	<1.0	negative ^3^	
milk product 4	sheep milk	sheep	95.4	positive ^3^	
		goat	4.5	positive ^3^	not declared, 1%–5%

-: Not detected, r.s.: ratio suspicious. ^1^ Relative quantification based on normalization. ^2^ Relative quantification by using a reference real-time PCR assay. ^3^ Obtained by the DNA array. ^4^ Sum of pork and wild boar. ^5^ In most cases species of casings are not detectable by DNA-based methods.

**Table 3:** Results for pet food products.

		Result			
Sample	Animal Species Declared	Animal Species Detected	DNA Metabarcoding Ratio of Reads (%)	Real-Time PCR (Ratio of DNA (%)) or DNA Array (Positive/Negative)	Comment
1	65% deer (heart, liver, lung, rumen)	red deer	96.3	92.9 ^1^	
		pork	1.7	<1.0 ^1^	not declared, 1%–5%
		fallow deer	-	6.0 ^1^	
		sheep	<1.0	<1.0 ^1^	
		chicken	<1.0	<1.0 ^1^	
		cattle	<1.0	<1.0 ^1^	
		kangaroo	<1.0	positive ^2^	
2	60% deer meat	pork	47.1	32.6 ^1^	not declared, >5%
		roe deer	36.0	55.0 ^1^	
		red deer	16.9	12.4 ^1^	
3	51% deer meat, <2.5% chicken liver	red deer	96.2	95.9 ^1^	
		roe deer	2.4	3.1 ^1^	
		pork	1.0	<1.0 ^1^	not declared, 1%–5%
		rabbit	<1.0	positive ^2^	
		chicken	negative	negative ^1^	declared, but not detected
4	59% fresh meat from deer and roe deer, 1.2% eggshell powder	red deer	62.4	53.0 ^1^	
		mallard	29.8	positive ^2^	not declared, >5%
		chicken	6.5	16.9 ^1^	
		fallow deer	-	2.3 ^1^	
		roe deer	<1.0	<1.0 ^1^	declared and detected, r.s.
		pork, sheep, cattle	<1.0	<1.0 ^1^	
5	10% deer meat (dried and ground)	chicken	38.1	25.7 ^1^	not declared, >5%
		turkey	12.3	7.3 ^1^	not declared, >5%
		mallard	10.7	positive ^2^	not declared, >5%
		horse	33.0	15.8 (Equidae) ^1^	not declared, >5%
		Muscovy duck	4.6	positive ^2^	not declared, 1%–5%
		donkey	1.1	positive ^2^	not declared, 1%–5%
		cattle	<1.0	<1.0 ^1^	
		deer	negative	negative ^1, 2^	declared, but not detected
6	28% fresh and 26% dried deer meat, 9% chicken fat, 2% dried eggs, 2% fresh and 2% dried herrings, 1% fish oil	pork	92.2	39.4 ^1^	not declared, >5%
		fish	-	positive ^2^	
		chicken	3.9	36.0 ^1^	
		red deer	2.7	2.3 ^1^	declared and detected, r.s.
		turkey	<1.0	22.0 **^1^**	
		sheep	<1.0	<1.0 ^1^	
			
			
7	18% dried Muscovy duck meat, 9.4% dried and ground deer meat, 6.3% dried whiting, 6.3% ground wild bones, egg yolk powder	cattle	67.8	59.9 ^1^	not declared, >5%
		chicken	9.7	13.3 ^1^	not declared, >5%
		mallard	7.1	positive ^2^	not declared, >5%
		red deer	7.5	2.8 ^1^	
		turkey	5.0	2.3 ^1^	not declared, >5%
		Muscovy duck	1.8	positive ^2^	declared and detected, r.s.
		sheep	<1.0	<1.0 ^1^	
		sika deer	<1.0	-	
		goat	<1.0	positive ^2^	
		fish	-	positive^2^	
8	50% meat and animal byproducts, 4% ostrich and deer	pork	52.4	3.8 ^1^	
		cattle	30.5	69.6 ^1^	
		chicken	16.8	26.5 ^1^	
		turkey	<1.0	<1.0 ^1^	
		mallard	<1.0	negative ^2^	
		ostrich, deer	negative	negative ^2^	declared, but not detected
9	35% cattle, 31% poultry, 4% deer	cattle	71.4	43.6 ^1^	
		turkey	9.0	8.3 ^1^	
		reindeer	12.3	positive ^2^	
		chicken	6.0	6.4 ^1^	
		mallard	<1.0	positive ^2^	
		pork, sheep, horse	<1.0	<1.0 ^1^	
		red deer	negative	negative ^1^	
10	lung, meat, kidney, liver, udder, 5% deer	pork	89.3	88.8 ^1^	
		cattle	9.7	10.5 ^1^	
		red deer	<1.0	<1.0 ^1^	declared and detected, r.s.
		chicken	<1.0	<1.0 ^1^	
11	48% fresh deer meat, 4% entrails of deer	mallard	96.3	31.0 ^1^	not declared, >5%
		goat	1.7	<1.0 ^1^	not declared, 1%–5%
		turkey, chicken	<1.0	<1.0 ^1^	
		pork, sheep	<1.0	<1.0 ^1^	
		deer	negative	negative ^1^	declared, but not detected
12	50% roe deer (60% meat, 25% heart, 10% lung, 5% liver)	red deer	98.1	97.9 ^1^	not declared, >5%
		horse	1.6	<1.0 (Equidae) ^1^	not declared, 1%–5%
		cattle	<1.0	1.7 ^1^	
		fallow deer	-	<1.0 ^1^	
		roe deer	negative	negative ^1^	declared, but not detected
13	99% deer meat	chicken	71.4	51.8 ^1^	not declared, >5%
		kangaroo	17.6	positive ^2^	not declared, >5%
		red deer	10.3	3.8 ^1^	declared and detected, r.s.
		rabbit	<1.0	positive ^2^	
		pork, cattle	<1.0	<1.0 ^1^	
14	75% deer (meat, heart, lung)	pork	84.3	45.1 ^1^	not declared, >5%
		cattle	6.4	10.0 ^1^	not declared, >5%
		roe deer	4.2	38.9 ^1^	
		mallard	2.2	1.5 ^1^	not declared, 1%–5%
		turkey	1.4	<1.0 ^1^	not declared, 1%–5%
		red deer	1.5	1.7 ^1^	
15	100% deer meat	roe deer	65.8	12.6 ^1^	
		cattle	31.2	87.0 ^1^	not declared, >5%
		chicken	<1.0	<1.0 ^1^	
		pork	1.9	<1.0 ^1^	not declared, 1%–5%
		red deer	1.0	<1.0 ^1^	
16	50% roe deer	turkey	98.2	99.6 ^1^	not declared, >5%
		red deer, horse	<1.0	<1.0 ^1^	
		pork	<1.0	<1.0 ^1^	
		roe deer	negative	negative ^1^	declared, but not detected
17	60% deer	red deer	40.4	25.3 ^1^	
		cattle	36.3	73.0 ^1^	not declared, >5%
		pork	22.9	1.7 ^1^	not declared, >5%
		chicken	<1.0	<0.1 ^1^	
18	46% poultry meat, 8% roe deer	chicken	55.7	83.8 ^1^	
		turkey	26.7	13.0 ^1^	
		sika deer	16.6	-	not declared, >5%
		cattle	<1.0	<1.0 ^1^	
		red deer, pork	<1.0	<1.0 ^1^	
		fallow deer	-	3.0 ^1^	
		roe deer	negative	negative ^1^	declared, but not detected
19	51% meat and animal byproducts, 12% chicken, turkey, duck	pork	58.5	55.0 ^1^	
		chicken	26.1	28.4 ^1^	
		turkey	9.0	10.1 ^1^	
		cattle	5.3	4.6 ^1^	
		mallard	<1.0	<1.0 ^1^	
		guinea fowl	<1.0	<1.0 ^1^	
20	51% meat and animal byproducts, 12% cattle, sheep, chicken	chicken	45.2	53.6 ^1^	
		pork	40.2	15.3 ^1^	
		cattle	10.9	26.8 ^1^	
		sheep	3.5	4.3 ^1^	
		turkey	<1.0	positive ^2^	
21	33% meat and animal byproducts, 4% poultry, 4% deer	pork	94.4	83.6 ^1^	
		chicken	3.9	15.3 ^1^	
		guinea fowl	<1.0	<1.0 ^1^	
		turkey	<1.0	1.0 ^1^	
		deer	negative	negative ^2^	declared, but not detected
22	meat and animal byproducts (4% turkey, 4% duck, 4% game)	chicken	49.4	57.6 ^1^	
		pork	25.1	13.4 ^1^	
		cattle	12.6	7.3 ^1^	
		turkey	6.3	19.5 ^1^	
		duck	4.1	<1.0 ^1^	
		sheep	1.9	<1.0 ^1^	
		horse	<1.0	<1.0 (Equidae) ^1^	
		fish	-	positive ^2^	
		game	negative	negative ^2^	declared, but not detected
23	40% chicken (heart, meat, liver, stomachs, necks), 28.7% broth, 28% rabbit	chicken	99.1	positive ^2^	
		cattle	<1.0	positive ^2^	
		rabbit	<1.0	positive ^2^	declared and detected, r.s.
			
			

-: Not detected, r.s.: ratio suspicious. ^1^ Relative quantification by using a reference real-time PCR assay. ^2^ Obtained with the DNA array.

## Data Availability

The data presented in this study are available on request.

## References

[1] Ballin N. (2010). Authentication of meat and meat products. Meat Sci..

[2] Montowska M., Pospiech E. (2010). Authenticity Determination of Meat and Meat Products on the Protein and DNA Basis. Food Rev. Int..

[3] Ballin N.Z., Vogensen F., Karlsson A.H. (2009). Species determination—Can we detect and quantify meat adulteration?. Meat Sci..

[4] Kumar A., Kumar R.R., Sharma B.D., Gokulakrishnan P., Mendiratta S.K., Sharma D. (2013). Identification of Species Origin of Meat and Meat Products on the DNA Basis: A Review. Crit. Rev. Food Sci. Nutr..

[5] Amaral J., Meira L., Oliveira B., Mafra I. (2016). Advances in Authenticity Testing for Meat Speciation. Advances in Food Authenticity Testing.

[6] Lo Y.-T., Shaw P.-C. (2018). DNA-based techniques for authentication of processed food and food supplements. Food Chem..

[7] Iwobi A.N., Huber I., Hauner G., Miller A., Busch U. (2010). Biochip Technology for the Detection of Animal Species in Meat Products. Food Anal. Methods.

[8] (2019). ISO 20813: 2019—Molecular Biomarker Analysis—Methods of Analysis for the Detection and Identification of Animal Species in Foods and Food Products (Nucleic Acid-Based Methods)—General Requirements and Definitions.

[9] Eugster A., Ruf J., Rentsch J., Hübner P., Köppel R. (2007). Quantification of beef and pork fraction in sausages by real-time PCR analysis: Results of an interlaboratory trial. Eur. Food Res. Technol..

[10] Eugster A., Ruf J., Rentsch J., Köppel R. (2009). Quantification of beef, pork, chicken and turkey proportions in sausages: Use of matrix-adapted standards and comparison of single versus multiplex PCR in an interlaboratory trial. Eur. Food Res. Technol..

[11] Köppel R., Ruf J., Rentsch J. (2011). Multiplex real-time PCR for the detection and quantification of DNA from beef, pork, horse and sheep. Eur. Food Res. Technol..

[12] Köppel R., Eugster A., Ruf J., Rentsch J. (2012). Quantification of Meat Proportions by Measuring DNA Contents in Raw and Boiled Sausages Using Matrix-Adapted Calibrators and Multiplex Real-Time PCR. J. AOAC Int..

[13] Laube I., Zagon J., Spiegelberg A., Butschke A., Kroh L.W., Broll H. (2007). Development and design of a ’ready-to-use’ reaction plate for a PCR-based simultaneous detection of animal species used in foods. Int. J. Food Sci. Technol..

[14] Druml B., Kaltenbrunner M., Hochegger R., Cichna-Markl M. (2016). A novel reference real-time PCR assay for the relative quantification of (game) meat species in raw and heat-processed food. Food Control.

[15] Iwobi A., Sebah D., Spielmann G., Maggipinto M., Schrempp M., Kraemer I., Gerdes L., Busch U., Huber I. (2017). A multiplex real-time PCR method for the quantitative determination of equine (horse) fractions in meat products. Food Control.

[16] Köppel R., Ruf J., Zimmerli F., Breitenmoser A. (2008). Multiplex real-time PCR for the detection and quantification of DNA from beef, pork, chicken and turkey. Eur. Food Res. Technol..

[17] Kaltenbrunner M., Hochegger R., Cichna-Markl M. (2018). Tetraplex real-time PCR assay for the simultaneous identification and quantification of roe deer, red deer, fallow deer and sika deer for deer meat authentication. Food Chem..

[18] Dolch K., Andrée S., Schwägele F. (2020). Comparison of Real-Time PCR Quantification Methods in the Identification of Poultry Species in Meat Products. Foods.

[19] Staats M., Arulandhu A.J., Gravendeel B., Holst-Jensen A., Scholtens I., Peelen T., Prins T.W., Kok E. (2016). Advances in DNA metabarcoding for food and wildlife forensic species identification. Anal. Bioanal. Chem..

[20] Fernandes T.J.R., Amaral J.S., Mafra I. (2020). DNA barcode markers applied to seafood authentication: An updated review. Crit. Rev. Food Sci. Nutr..

[21] Franco C.M., Ambrosio R.L., Cepeda A., Anastasio A. (2021). Fish intended for human consumption: From DNA barcoding to a next-generation sequencing (NGS)-based approach. Curr. Opin. Food Sci..

[22] Nehal N., Choudhary B., Nagpure A., Gupta R.K. (2021). DNA barcoding: A modern age tool for detection of adulteration in food. Crit. Rev. Biotechnol..

[23] Cottenet G., Blancpain C., Chuah P.F., Cavin C. (2020). Evaluation and application of a next generation sequencing approach for meat species identification. Food Control.

[24] Druml B., Cichna-Markl M. (2014). High resolution melting (HRM) analysis of DNA—Its role and potential in food analysis. Food Chem..

[25] López-Oceja A., Nuñez C., Baeta M., Gamarra D., de Pancorbo M. (2017). Species identification in meat products: A new screening method based on high resolution melting analysis of cyt b gene. Food Chem..

[26] Parvathy V.A., Swetha V.P., Sheeja T.E., Leela N.K., Chempakam B., Sasikumar B. (2014). DNA Barcoding to Detect Chilli Adulteration in Traded Black Pepper Powder. Food Biotechnol..

[27] Chin T.C., Adibah A., Hariz Z.D., Azizah M.S. (2016). Detection of mislabelled seafood products in Malaysia by DNA barcoding: Improving transparency in food market. Food Control.

[28] Dobrovolny S., Blaschitz M., Weinmaier T., Pechatschek J., Cichna-Markl M., Indra A., Hufnagl P., Hochegger R. (2019). Development of a DNA metabarcoding method for the identification of fifteen mammalian and six poultry species in food. Food Chem..

[29] Martin M. (2011). Cutadapt Removes Adapter Sequences from High-Throughput Sequencing Reads. EMBnet J..

[30] Edgar R.C. (2010). Search and clustering orders of magnitude faster than BLAST. Bioinformatics.

[31] Rentsch J., Weibel S., Ruf J., Eugster A., Beck K., Köppel R. (2012). Interlaboratory validation of two multiplex quantitative real-time PCR methods to determine species DNA of cow, sheep and goat as a measure of milk proportions in cheese. Eur. Food Res. Technol..

[32] Köppel R., Daniels M., Felderer N., Brünen-Nieweler C. (2013). Multiplex real-time PCR for the detection and quantification of DNA from duck, goose, chicken, turkey and pork. Eur. Food Res. Technol..

[33] Druml B., Mayer W., Cichna-Markl M., Hochegger R. (2015). Development and validation of a TaqMan real-time PCR assay for the identification and quantification of roe deer (*Capreolus capreolus*) in food to detect food adulteration. Food Chem..

[34] Kaltenbrunner M., Hochegger R., Cichna-Markl M. (2018). Red deer (*Cervus elaphus*)-specific real-time PCR assay for the detection of food adulteration. Food Control.

[35] Kaltenbrunner M., Hochegger R., Cichna-Markl M. (2018). Development and validation of a fallow deer (Dama dama)-specific TaqMan real-time PCR assay for the detection of food adulteration. Food Chem..

[36] Evaluation Report Proficiency TestDLA 45/2019 Animal Species-Screening III: Buffalo Milk, Cow’s Milk, Sheep’s Milk and Goat’s Milk in Dairy Product (Herder Cheese) (2020) DLA—Proficiency Tests GmbH. http://www.dla-lvu.de/Auswerteberichte%202019/PT%20-%20DLA%2045-2019%20Final%20Report%20Animal%20Species-Screening%20III.pdf.

[37] Pirondini A., Bonas U., Maestri E., Visioli G., Marmiroli M., Marmiroli N. (2010). Yield and amplificability of different DNA extraction procedures for traceability in the dairy food chain. Food Control.

[38] Kaltenbrunner M., Mayer W., Kerkhoff K., Epp R., Rüggeberg H., Hochegger R., Cichna-Markl M. (2020). Applicability of a duplex and four singleplex real-time PCR assays for the qualitative and quantitative determination of wild boar and domestic pig meat in processed food products. Sci. Rep..

[39] Goedbloed D.J., Megens H., Van Hooft P., Herrero-Medrano J.M., Lutz W., Alexandri P., Crooijmans R.P.M.A., Groenen M., Van Wieren S.E., Ydenberg R.C. (2013). Genome-wide single nucleotide polymorphism analysis reveals recent genetic introgression from domestic pigs into Northwest European wild boar populations. Mol. Ecol..

[40] Dzialuk A., Zastempowska E., Skórzewski R., Twarużek M., Grajewski J. (2017). High domestic pig contribution to the local gene pool of free-living European wild boar: A case study in Poland. Mammal Res..

[41] Böhme K., Calo-Mata P., Barros-Velázquez J., Ortea I. (2019). Review of Recent DNA-Based Methods for Main Food-Authentication Topics. J. Agric. Food Chem..

